# Covering Aluminum Oxide Nanoparticles with Biocompatible Materials to Efficiently Deliver Subunit Vaccines

**DOI:** 10.3390/vaccines7020052

**Published:** 2019-06-17

**Authors:** Ning Wang, Changlu Qiu, Minnan Chen, Ting Liu, Ting Wang

**Affiliations:** 1 School of Food and Bioengineering, Hefei University of Technology, 193 Tun Brook Road, Hefei 230009, Anhui, China; nwangcn@hfut.edu.cn; 2 School of Pharmacy, Anhui Medical University, 81 Plum Hill Road, Hefei 230032, Anhui, China; clqiucn@163.com (C.Q.); mnchencn@163.com (M.C.); tingliucn@163.com (T.L.)

**Keywords:** alum adjuvant, cutaneous immunization, humoral immunoresponse, cellular immunity, lymph node, antigen cross-presentation

## Abstract

Subunit vaccines have advantages of good safety, minimal reactogenicity, and high specificity. However, subunit vaccines also show a crucial disadvantage of poor immunogenicity and, therefore, are often formulated with an adjuvant carrier to form a vaccine adjuvant-delivery system (VADS) to enhance their efficacies. Alums, the coarse aggregates of the insoluble aluminum salts, are the conventional adjuvants and have been widely used in clinical vaccines for a long time. Unfortunately, alums also show two main drawbacks of low potency in eliciting cellular immunity, and high reactogenicity to cause unwanted inflammations. Therefore, herein the phospholipid bilayer-coated aluminum oxide nanoparticles (PLANs) and the PEGylated PLANs (PEG-PLANs) were engineered as a VADS to overcome the drawbacks of both subunit vaccines and coarse alums, while synergizing their functions. In vitro experiments demonstrated that, unlike the micron-sized alums, the nanosized PLANs and PEG-PLANs loaded with model antigen of ovalbumin (OVA) showed a high safety profile and were able to promote APC (antigen-presenting cell) uptake and engender lysosome escape for enhancing the MHC (major histocompatibility complex)-I-antigen display. Subcutaneously administered to mice, PLANs and, especially, PEG-PLANs smoothly trafficked into the draining lymph nodes, wherein the densely clustered immune cells were activated in substantial numbers, leading to robust immunoresponses and efficient production of the anti-antigen antibodies and CD8+ T cells. Thus, the aluminum-based nanocarriers, especially the PEG-PLANs, are a promising VADS possessing the potential of eliciting strong and comprehensive immunity against pathogens.

## 1. Introduction

Vaccination is a cost-efficient strategy to control various diseases such as autoimmunity, anaphylaxis, cancer, and especially infections. This is exemplified by the remarkable reduction in the mortality of epidemic outbreaks in recent years and the prolonged life-span of people as a result of prevalent vaccination [1].

The conventional vaccines are made of the inactivated or live attenuated whole microbes and can elicit strong immunoresponses, but are also argued to be associated with certain safety concerns. These concerns on whole microbe-based vaccines arise from their potential of reversion of virulence or induction of the off-target immunity to cause unwanted side effects while weakening vaccination effects [2,3]. Therefore, subunit vaccines, which contain only the purified antigens (Ags), were timely developed at the beginning of 1970s when the genetic reassortment of virus strains rapidly matured and was innovatively introduced into the production of vaccines [4]. Nowadays, subunit vaccines have received more and more research interests owing to their good reputation in inducing the accurate immunoresponses while causing minimal safety concerns. In addition, subunit vaccines are sharing the advantage of flexibility in formulation with other bioactive ingredients or combination with a carrier to boost immunostimulatory activities [5]. However, subunit vaccines often show a crucial drawback of poor immunogenicity owing to lack of other microbial components, including especially the ones bearing the intrinsic adjuvanticity. To compensate for this defect, subunit vaccines are often combined with an adjuvant or engineered with proper excipients to form a carrier engendering vaccine adjuvant-delivery system (VADS) to enhance efficacy [6,7].

Alums (insoluble aluminum salts) are a conventional adjuvant or VADS that has been widely used for nearly a century to enhance the efficacy of numerous inactivated or subunit vaccines with unknown mechanisms [2]. As a classical vaccine adjuvant, micron-sized alums, such as Al(OH)_3_, AlPO_4_, and (Al)_2_(SO_4_)_3_, usually exist in the form of gel-like aggregates constituted of the sub-10-nm-sized crystals of aluminum (Al) salts and thus, vary remarkably in shape and size. Alums are known to induce potent humoral immunity with high levels of the anti-Ag Abs (antibodies). But alums are also known of the impotency in triggering cellular immunoresponses and can rarely induce effective anti-Ag CTLs (cytotoxic T lymphocytes), which, however, are one of the necessary elements for erasing certain pathogens, such as the intracellular microbes and cancer cells [8]. In addition, alums in the form of the micron-sized coarse aggregates show relatively high reactogenicity and sometimes cause local inflammatory effects and even severe reactions in recipients [9]. Therefore, in the past years alums have been reformulated to convert the coarse gel-like forms into certain new modalities, such as nanoparticles (NPs) [10], nanorods [11], and the complex of alum-TLR4a (Toll-like receptor 4 agonist) MPLA (monophosphoryl lipid A) [12]. Surprisingly, it was recently reported that injection of alum with a carefully optimized protocol and a precise vaccination time schedule, induced effective anti-tumor CTL responses showing non-specific tumor suppression [13]. These outcomes indicate that innovative strategies may remarkably improve adjuvanticity of alums and even enable the alum-adjuvanted vaccines to elicit both humoral and cellular immunity, suggesting the Al-based adjuvants of more potentials in boosting immunoresponse than those in practical use [14].

Recently, based on the phosphophilicity of aluminum [15], we have successfully engineered the phospholipid bilayer-coated aluminum NPs (PLANs) as a novel type of VADS that proves able to stimulate strong humoral and even cellular immunoresponses against Ags [16]. However, in that study, PLANs were made with the unstable aluminum hydroxide (Al(OH)_3_) NPs obtained by neutralizing in a solution of aluminum chloride (AlCl_3_) with alkaline sodium hydroxide (NaOH). The prepared Al(OH)_3_) NPs demonstrated a high heterogenicity in size and morphology, significantly lowering the batch-to-batch consistency and thus reproducibility of the final product PLANs [9]. Although the instability and heterogenicity of the Al(OH)_3_-based PLANs were improved through using a procedure of reverse ethanol injection-lyophilization (REIL), the engagement of organic solvent in the preparation introduced unbeneficial factors with the potential to compromise the activity of Ags of interest.

In this report, the Ag-loaded PLANs and PEGylated-PLANs (PEG-PLANs) as a novel VADS were engineered using the stable aluminum oxide (Al_2_O_3_) NPs (ANs) instead of the unstable Al(OH)_3_ NPs while employing the thin film-rehydration procedure in preparation. This classical procedure is of no novelty but allows the labile Ags to avoid the contact with the organic solvent, thus maintaining well their immunostimulatory activity. In addition, the comparative studies were performed on AMs (aluminum microparticles), ANs, PLANs, and PEG-PLANs to investigate their special abilities in triggering the anti-Ag immunoresponses through in vitro and in vivo experiments.

## 2. Materials and Methods

### 2.1. Materials

SPC (soy phosphatidylcholine), DPPC (1,2-dipalmitoyl-sn-glycero-3-phosphocholine), and DSPE-PEG_2000_ (1,2-distearoyl-sn-glycero-3-phosphoethanolamine-*N*-(methoxy(polyethylene glycol)_2000_) (ammonium salt)) were products by Avanti Polar Lipids (Alabaster, Alabama, USA). Aluminum oxide (Al_2_O_3_) nanoparticles with a size of 40 nm, ovalbumin (OVA), bovine serum albumin (BSA), calcein, 4′,6-diamidino-2-phenylindole (DAPI), 3-(4,5-dimethylthiazol-2-yl)-2,5-diphenyltetrazolium bromide (MTT), CFSE (5(6)-carboxyfluorescein diacetate N-succinimidyl ester), and 3,3,5-tetramethylbenzidine (TMB) were products by Sigma-Aldrich (Merck, Darmstadt, Germany). Goat anti-mouse IgG-horse radish peroxidase (HRP), IgG1-HRP, IgG2a-HRP, and mouse cytokines IFN-γ and IL-4 ELISA assay kits, fluorochrome-labeled anti-mouse Abs against different cell surface antigens such as CD40, CD80, and CD86 for APC activation assay, and CD4 and CD8 for T lymphocytes activation assay were purchased from either eBioscience (San Diego, USA) or BioLegend (San Diego, USA). LysoTracker^®^-red, HyClone RPMI 1640, DMEM/F12 cell culture medium were purchased from Thermo Fisher Scientific Inc. (Waltham, AMs, USA). Chromatographic grade solvents, analytic grade agents, and other chemicals were provided by Sinopharm Chemical Reagent Co., Ltd. (Beijing, China). Pure water was produced using Milli-Q^®^ IQ 7000 Ultrapure Water System (Merck, Germany).

### 2.2. Preparation of Al-Based Carriers

The blank or cargo-loaded Al-based nanocarriers including PLANs and PEG-PLANs were prepared by thin film-rehydration procedure. Briefly, in a pear-shaped flask, SPC was dissolved in chloroform, which was evaporated at room temperature under a reduced pressure using a rotary evaporator and completely removed in a vacuum chamber overnight to form a thin lipid film. Then an aqueous suspension of (OVA- or calcein-associated) ANs was added into the flask for rehydration of the lipid film at an electromagnetic stir of 360 rpm (round per min) at 45 °C for 2 h under N_2_ protection. Then, the rehydrated samples were washed thrice with saline to remove the excess empty liposomes and free cargos by centrifugation at 2000 rpm for 15 min at 4 °C. The proper ratio of phospholipid to ANs was estimated according to our previous report [16], while phospholipids of empty liposomes were quantified by the high performance liquid chromatography (HPLC) method described in the Characterization section.

For preparation of the PEG-PLANs, a procedure of post insertion was used [17]. Briefly, an appropriate amount of DSPE-PEG_2000_ (with the mole ratio of SPC/DSPE-PEG_2000_ = 20:1) was dissolved in chloroform in a 5-mL ampoule. Then the samples were removed of organic solvent by nitrogen gas flow to form a thin film, which was subsequently dried in a vacuum chamber overnight. Finally, the thin film of DSPE-PEG_2000_ was rehydrated with an aqueous dispersion of PLANs at a stir of 120 rpm at 45 °C for 1 h under N_2_ protection, thus forming PEG-PLANs.

Calcein, which at a neutral pH condition is a membrane impermeable fluorescent agent with a self-quenched concentration of >4 mM [18], was complexed (at 0.01 mM) with Al particles before lipid coating to label the prepared carriers. The labeled carriers were used for inspecting APC uptake, intracellular location, and in vivo traffic of Al-based VADSs by either a LSCM (laser scanning confocal microscopy) (Leica TCS SP5, Wetzlar, Germany) or flow cytometry (BD FACSVerse™, San Jose, CA, USA).

Aluminum phosphate (AlPO_4_) crystal salt was smashed into AlPO_4_ microparticles (AMs) with a size of around 2 μm and was used as a control, i.e., as the conventional adjuvant alum.

### 2.3. Characterization of the Al-Based Nanocarriers

The nanoparticulate samples were characterized of size and zeta potential by DLS (dynamic light scattering) and ELS (electrophoretic light scattering), respectively. A Malvern ZS90 Zetasizer (Malvern, Worcestershire, UK) with a 633 nm-wavelength He–Ne laser was used to collect the optics data with the detector at 90° to incident light at 25 °C.

FTIR (Fourier transform infrared spectroscopy) spectra of the lyophilized PLANs, anhydrous AlPO_4_, and SPC were obtained for dissection of the bond interaction between aluminum and phosphate group of SPC. FTIR detection was carried out by transmission method, using the 2 mg sample dispersed in a 200 mg KBr disk, in a FTIR spectrophotometer (IR Prestige-21, Shimadzu, Japan).

The Al-based nanocarriers were imaged using a TEM (transmission electron microscope) system (HF-3300 TEM System, Hitachi Ltd., Tokyo, Japan), and the samples were negatively stained with uranyl, according to previous reports [16,19].

Phospholipids were quantitated with a HPLC (high performance liquid chromatography) system (LC-6AD liquid chromatograph, SPD-20A UV detector, SIL-10AF autosampler, Shimadzu) equipped with ODS C18 column (5 µm, 4.6 × 150 mm). The SPC in empty liposomes was isolated from PLANs or PEG-PLANs by centrifugation at 5000× *g* for 10 min at 4 °C and was quantified by HPLC method according to a previous report but with a little modification [20]. The UV detection wavelength was 205 nm and a mobile phase of methanol-isopropanol-water-trifluoroacetic acid (95:5:100:0.05, *v*/*v*) with flow rate of 1 mL/min at 30 °C.

The Ag association efficiency of samples was calculated according to the ratio of the carrier-associated Ag to total Ag. The carrier-associated Ag was deduced from subtraction of the total Ag with free Ag, which was isolated from sample by centrifugation at 10,000× *g* for 10 min at 4 °C and quantitated by Bradford assay.

For Ag release test, in triplicate, 0.5 mL of samples that had been removed of free Ag was added into a 10-mL conical flask with a ground stopper and diluted with the buffered solutions (pH 7.4 or 5.5) to 5 mL and incubated at 37 °C under stirring of 100 rpm. At different time intervals (0.5, 1, 2, 4, 8, 16, 24, 48 h), 30 μL of release medium was accurately taken out of the flask, which was immediately supplemented with an identical amount of blank medium. The sample-containing medium was centrifuged at 10,000× *g* for 10 min at 4 °C for isolation of the released Ags in the supernatant, which was transferred into a hole of a 96-well plate. Then the plate was stored at 4 °C in a refrigerator until the Ag assay, which was carried out with the micro-Bradford protocol, whereby the plate was read at 595 nm in a microplate reader (µQuant, BioTek Instruments, Inc., Vermont, USA) [21,22].

### 2.4. APC Uptake and Safety Evaluation of the Al-Based Carriers

The prepared Al-based carriers, including AMs, ANs, PLANs, and PEG-PLANs, were evaluated of APC uptake using mouse BMDCs (mouse bone marrow-derived dendritic cells), which were derived from mouse bone marrow precursors according to previous reports [23,24]. For APC uptake assay, the BMDCs were first transferred onto a 24-well plate with 1 mL of 10^5^ cells per well and incubated for 24 h in a cell culture chamber at 37 °C at an atmosphere of 5% CO_2_. Then, each well containing BMDCs was supplemented with 100 µL of free calcein or calcein-loaded carriers (AMs, ANs, PLANs and PEG-PLANs, at 10 µg/mL Al final concentration), and mixed homogeneously for another 4 h incubation in the cell culture chamber. After the incubation, the co-cultured cells were removed of the un-associated agents or nanocarriers through centrifugation at 800× *g* at 4 °C and thrice PBS washing. Finally, the cells were tested by flow cytometry (BD FACSVerse™, San Jose, CA, USA) and assayed with FlowJo software (Tree Star, Ashland, OR, USA) to evaluate cellular uptake of carriers. Also, a small fraction of cells was imaged by LSCM (with a laser scanning confocal microscope, Leica TCS SP5, Wetzlar, Germany).

The safety of the blank Al-based carriers was evaluated by testing their cytotoxicity with the classical MTT method. Briefly, 100 μL of 10^5^ mouse macrophages (RAW264.7) was seeded in each well of a 96-well plate and incubated in a complete growth medium DMEM for a 24-h culture in a cell chamber. Then, in triplicate, the plate wells were individually added with one type of the Al-carrier samples at a certain concentration (each sample with a series of concentration ranging from 0 to 200 μg/mL Al), followed by another 24 h incubation at 37 °C. After the incubation and removal of supernatant, each well of the plate was added with 200 μL of DMEM containing 1 mg/mL MTT and incubated at 37 °C for 4 h. Thereafter, the plate was again removed of the supernatant and supplemented with 200 μL of DMSO in each well for another incubation at 37 °C for 30 min under shaking. After that, the optical absorbance (OA) of each well of the plate was measured using μQuant microplate reader at 540 nm wavelength. The viability index (%) was used to evaluate the safety of the Al-based VADS and was calculated according to the following Equation (1):

Viability index (%) = (OA_MP+particles_ − OA_blank_)/(OA_MP+PBS_ − OA_blank_) × 100% (1)

### 2.5. APC Activation and Intracellular Localization of Al-Based Nanocarriers

For tracking the intracellular agents, a fraction of the BMDCs co-cultured with Al particles were transferred onto a 12-well plate (10^4^ cells/well) and incubated in pre-warmed RPMI 1640 medium (37 °C) containing 50 nM LysoTracker-red and DAPI to localize cellular lysosomes and nuclei, respectively. After washing thrice, BMDCs were observed in a LSCM to distinguish in cells the DAPI-stained blue nuclei, LysoTracker-red-labelled lysosomes, and the green domains (occupied by calcein-carriers). The separation of green-occupied domains from the red dots (LysoTracker-red-labelled lysosomes) is thought the evidence indicating that the carrier has engendered lysosome escape to the cargos [25].

The ability of OVA-loaded Al-based carriers (AMs, ANs, PLANs, and PEG-PLANs) to activate APCs for maturation was tested with BMDCs [26]. Briefly, the cells cultured in a 24-well plate (0.5 × 10^5^ cells/well) with 0.5 mL RPMI 1640 medium were fed with 50 µL of 20 µg soluble OVA or carrier-loaded OVA for 30 h stimulation. After refreshment of culture RPMI 1640 medium, BMDCs were exposed to the fluorochrome-conjugated Abs against CD40, CD80, and CD86, which are markers of maturation of the activated APCs.

### 2.6. Mouse Vaccination

Female Kunming mice, aged 8 weeks, were provided from the Experimental Animal Center of Anhui Medical University. Animal experiments were approved by the Animal Ethic Committee at Anhui Medical University and were performed in compliance with the Declaration of Helsinki for Care and Use of Laboratory Animals (approval number LLSC20170284).

Mice were divided into different groups (6 mice per group) and subcutaneously immunized only once for each group with free OVA, the OVA-loaded AM, ANs, PLANs, or PEG-PLANs at a dose of 10 µg OVA, regardless of their bodyweight. The subcutaneous injection site was kept at the lateral aspect of left thigh near the knee.

### 2.7. In Vivo Tracking of Nanocarriers

To track in vivo Al-based nanocarriers, the calcein-loaded samples (ANs, PLANs, PEG-PLANs) were subcutaneously injected into mice at the left thigh lateral aspect near the knee (*n* = 3). Next, 4, 8, and 16 h after vaccination, the inguinal LNs responsible for draining the hindlimb region were isolated from the vaccinated mice for further investigation. To observe regional distribution of fluorescent nanocarriers, mouse inguinal LNs (lymph nodes) were harvested for making the OCT (optimum cutting temperature) compound-embedded cryosections to be viewed using a fluorescence microscope (IX83, Olympus, Japan). For quantitative analysis of the uptake of Al-based nanocarriers by immune cells within LNs, inguinal LNs were harvested to isolate immunocytes, which were assayed by flow cytometry.

### 2.8. Assay of the Ag-Specific Immunity Elicited by Al-Based Carriers

Three weeks after immunization, blood samples were collected from the mice via the retro-orbital sinus [27], which were let to stand for 30 min for clotting and then serum was collected through centrifugation at 3000× *g* for 10 min at 4 °C. The serum samples were assayed of the Ag-specific Abs by the indirect ELISA protocol [28]. Briefly, a 96-well plate was coated with 100 µL of OVA (10 µg/mL) in carbonate buffer (15 mM Na_2_CO_3_, 35 mM NaHCO_3_, pH 9.6) per well and incubated at 4 °C overnight. Then, after washing thrice with 200 µL PBST (PBS containing 0.05% *v*/*v* Tween-20), each well of the plate was blocked with 200 µL of 1% *w*/*v* BSA-containing PBST and incubated at 37 °C for 1 h for covering non-specific binding sites. After washing thrice with PBST, each well of the plate was loaded with 100 μL serum sample and then, after serial dilution with PBST-BSA (PBST containing 0.1% *w*/*v* BSA), it was incubated at 37 °C for 1 h. After washing thrice with PBST, each well was added with 100 μL of the HRP-conjugated secondary antibodies diluted with PBST-BSA (1:5000) and then incubated at 37 °C for 1 h. After washing five times with PBST, each well was supplemented with 100 μL of TMB substrate solution (containing 1 mM TMB, 3 mM H_2_O_2_, and 0.2 mM TBABH (tetramethylammonium borohydride)) for 20 min color development. Finally, the reaction was terminated by 100 μL of 1 M H_2_SO_4_ solution. Then, immediately, the optical absorbance (OA) at 450 nm, with a reference wavelength at 570 nm, of each well was measured using an automated microplate reader (µQuant, BioTek Instruments, Inc., Vermont, USA).

The serum samples were also tested of cytokines including IL-4 and IFN-γ by sandwich enzyme immunoassay using ELISA assay kits.

### 2.9. Differentiation of T Lymphocytes in Spleen

Three weeks after immunization, under an aseptic operation, the mice were excised of spleens for separation of splenocytes, which were isolated according to a previous report [28]. A total of 500 µL of 5 × 10^5^ cells were added to each well of a 24-well plate and incubated in the presence of 2.5 µg/mL OVA for 72 h at 37 °C in a cell chamber containing 5% CO_2_. Thereafter, the plate was centrifuged at 1000× *g* for 10 min and the supernatants were carefully collected and stored at −20 °C for detection of cytokines IL-4 and IFN-γ using the sandwich enzyme immunoassay. Then, 500 µL of PRMI 1640 was added to each well and the plate was vortexed to resuspend cells, which were stained with anti-CD4-Ab-PE and anti-CD8-FITC at 4 °C for 2 h. Finally, the cells were assayed by flow cytometry for evaluation of CD4+ T lymphocytes and CD8+ CTLs generated by vaccination with different formulations.

### 2.10. Statistical Analysis

All results were given as mean ± SD (standard deviation). Statistical differences among multiple groups were analyzed with ANOVA, and Dunnett’s post hoc *t*-test was employed to analyze the differences between groups. And the analyses were conducted using SPSS software, and a *p* < 0.05 was considered of significance, though this value is argued more than often leading to “hyped claims” [29].

## 3. Results

### 3.1. Characteristics of the Al-Based Carriers

**Figure 1:**
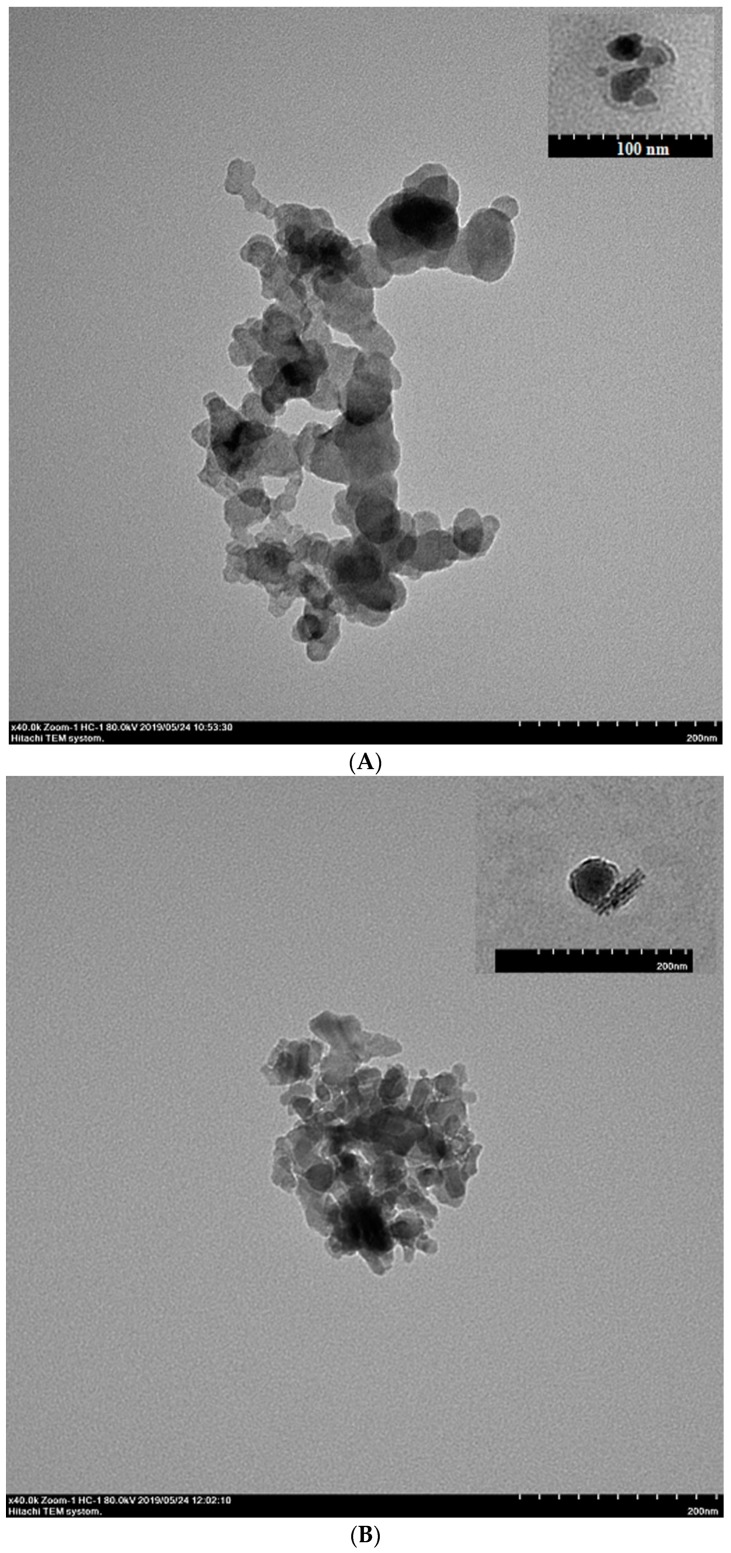

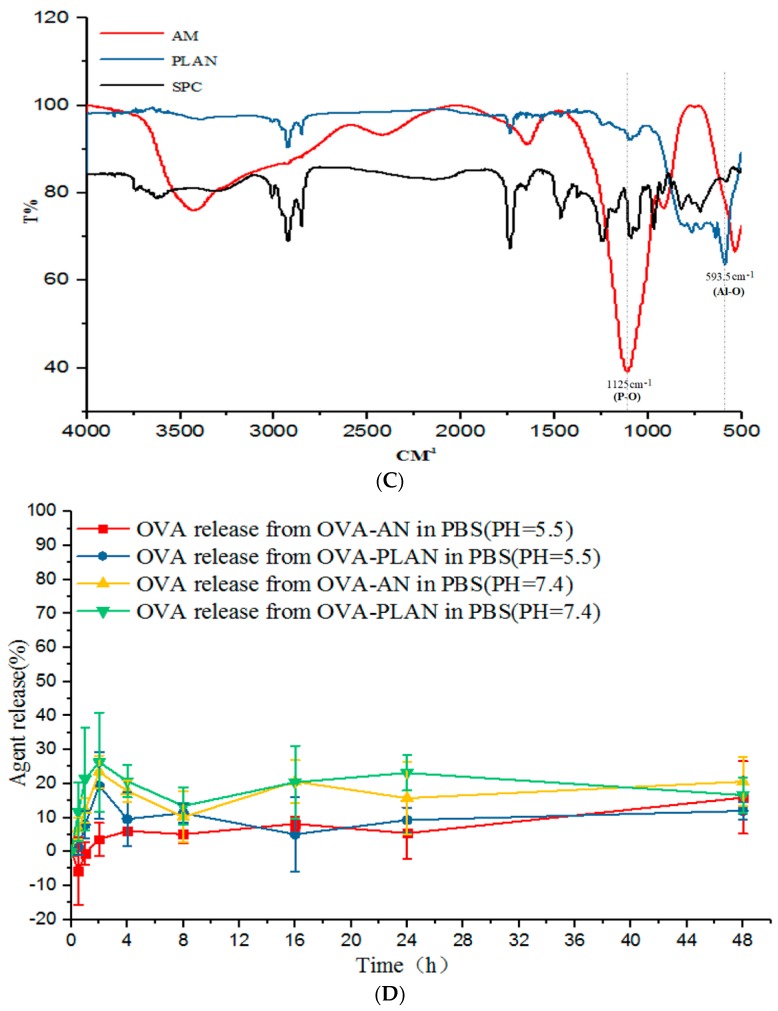
Features of the prepared Al-nanocarriers. (**A**) Transmission electron microscope (TEM) image of phospholipid bilayer-coated aluminum oxide nanoparticles (PLANs) displays the spherical shape and a size of 30 nm of the Al-nanocarriers (the inserted panel showing phospholipid coatings with thickness of around 5 nm observed with uranyl acetate negative staining). (**B**) TEM image of PEG-PLANs displays the spherical shape and a size of 30 nm of the Al-nanocarriers (the inserted panel showing phospholipid coatings with thickness of around 10 nm observed with uranyl acetate negative staining and the inexplicable presence of certain nano-sticks). (**C**) Fourier transform infrared spectroscopy (FTIR) spectra of aluminum microparticles (AMs) of Al(PO_4_)_3_, 1,2-dipalmitoyl-sn-glycero-3-phosphocholine (DPPC), and PLANs indicate, by comparative analysis, formation of covalent bonds between phosphate group and Al, as evidenced by a characteristic peak at ~1125 cm^−1^ which is resulted from the vibrations of Al–O–P. (**D**) Ovalbumin (OVA) release curves of the Al-based nanocarriers in different media at 37 °C showing trivial cargo release from these carriers (*n* = 3).

The hydrodynamic diameter, zeta potential (ζ), Ag association efficiency (AE) of ANs, PLANs, and PEG-PLANs are presented in Table 1. The size and ζ of ANs, PLANs, and PEG-PLANs were of around 30 nm and −30 mv, respectively. Association efficiency (AE) for OVA of ANs was 78% and did not change after coating with phospholipids and PEGylation. The TEM images indicated that most of the PLANs and PEG-PLANs maintained their spherical shape and a size of around 30 nm (Figure 1A,B), both of which were similar to those of the ANs (ANs were commercial products by Sigma with product number 642991, a representative image is presented on Sigma website). Notably, the thickness of the phospholipid coatings wrapping the PLANs or PEG-PLANs is of around 4 or 10 nm, suggesting that both types of Al-based NPs were covered with just one or two phospholipid bilayers (inserted panels in Figure 1A,B). It should be pointed out that, with unknown reason, certain nano-sticks were also noticed, when PLANs and, especially, PEG-PLANs were observed under a TEM apparatus using the uranyl acetate negative-staining protocol.

The FTIR (Fourier transform infrared spectroscopy) spectra demonstrated that phospholipid bilayers might be coated with Al_2_O_3_ NPs through formation of the Al–O–P covalent bond between phosphate group and aluminum (Figure 1C), as judged by characteristic peaks at ~1125 cm^−1^ and −593 cm^−1^ (Figure 1C), which are attributed to the vibrations of Al–O and P–O, respectively [16].

HPLC quantification (standard curve of Area_peak_ = 12017C_lipid_ + 6883.6, C: lipid concentration of μg/mL, R^2^ = 0.9911) confirmed that the phospholipids (DPPC) used for preparation of PLANs should satisfy the mole ratio of DPPC/AN of about 1:3. This was estimated by HPLC results, which showed that when the amount of phospholipid at mole ratio of DPPC/AN of 1:3 was used for preparation of PLANs, less than 8.3% (RSD = 0.17, *n* = 3) of total lipid was tested in empty liposomes which were separated by centrifugation at 2000× *g* for 15 min at 4 °C. The results were in agreement with our previous report [16].

The Ag release of the Al-based carriers was rather slow, since at 37 °C only less than 30% of the total Ag was released in 48 h, in a neutral (pH 7.4) or acidic medium (pH 5.5) (Figure 1D). Also, the Ag release from the Al-based carriers was not affected by the phospholipid coating or PEGylation, as shown in Figure 1D. Notably, after showing a small burst release at 0.5 h, the OVA release curve remains in a fluctuated horizon, indicating the release does not continue after 0.5 h. A possible explanation for this may lie in the fact that the OVA loaded on the Al-based nanocarriers exists in two forms: The OVA molecules that are directly associated with the Al-based carriers; and the OVA aggregates that are formed as a result of moderate attraction among identical molecules. The OVA molecules are firmly associated with the carriers, perhaps, via covalent bonds between P groups in OVA and Al of the carrier and, thus, will not be released. By contrast, the OVA aggregates contain the loosely associated molecules that are responsible for the burst release in the first 0.5 h. Since OVA is a polymer with a large molecular weight of 45 kDa, the dynamic balance between its adsorption and desorption on the fraction of the carrier-bound OVA is rather slow, leading to heterogeneity in sampling, which may account for the release fluctuation.

### 3.2. The Al-Nanocarriers Facilitate APC Uptake of Their Carried Cargos While Showing No Cytotoxicity to APCs

**Figure 2:**
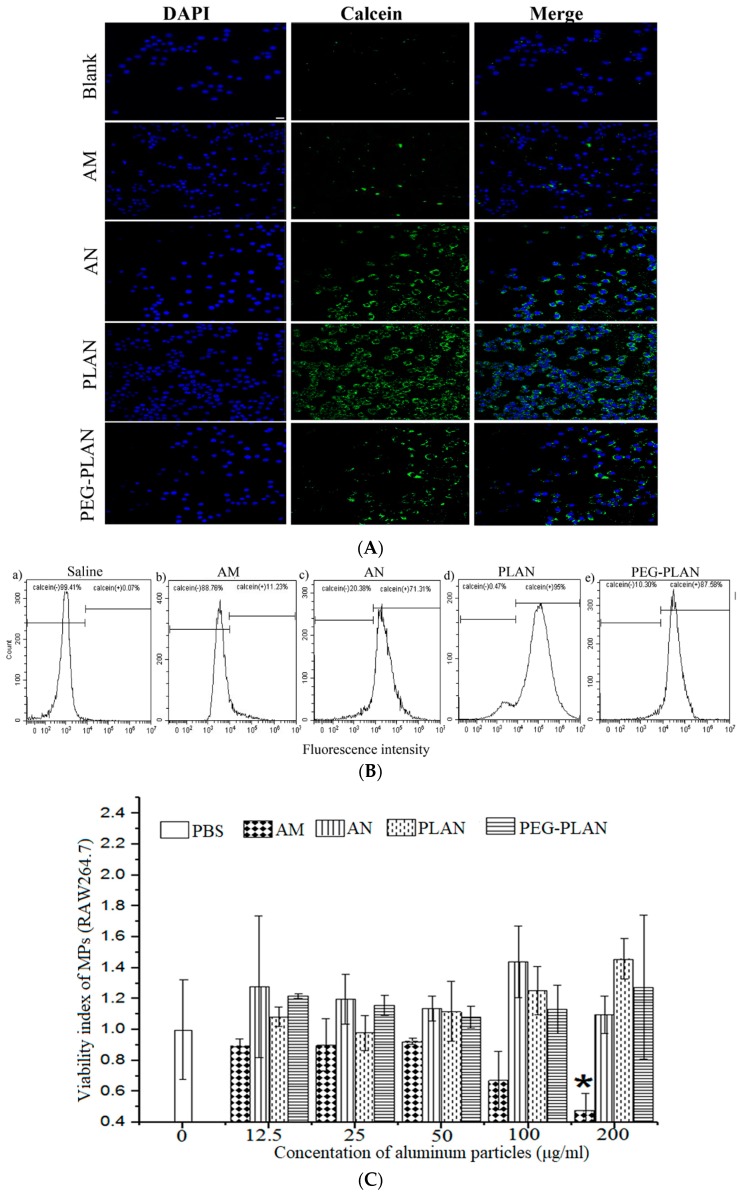
APC uptake and the cytotoxicity of the Al-based vaccine adjuvant-delivery systems (VADSs) (*n* = 3). (**A**) Fluorescent images showing mouse bone marrow-derived dendritic cells (BMDC) uptake of free calcein or the calcein-loaded Al-based carriers (bar = 10 μm, applicable to all panels). (**B**) The percentages of APCs that had internalized the Al-VADSs with the data gained by flow cytometry. (**C**) APC viability index derived from the 3-(4,5-dimethylthiazol-2-yl)-2,5-diphenyltetrazolium bromide (MTT) data obtained through incubation of murine MPs (macrophages) (RAW264.7) with different Al-VADSs.

To evaluate the ability of the Al-based carriers to facilitate APC uptake, the carriers were loaded with calcein and co-cultured in vitro at 37 °C with BMDCs, which were then observed by fluorescent LCSM and assayed by flow cytometry. Fluorescence images (Figure 2A) indicate that all the Al-based nanocarriers were remarkably internalized by BMDCs, while AM-carried or free agents were minimally taken up by BMDCs. These results were supported by the flow cytometry assay outcomes, which also show that the frequencies of APC uptake of different carriers ranked in the order of AMs < ANs < PEG-PLANs < PLANs. These results suggest that nanosization and phospholipid coating are efficient strategies to promote APC uptake (Figure 2B), whereas PEGylation may somewhat compromise this function.

The in vitro experiments also showed that the Al-based VADSs not only exhibited no cytotoxicity to murine macrophages (RAW264.7) but also showed growth promotion effects on this cell line, as indicated by the viability index values >1. By contrast, AM remarkably reduced cell viability, since it showed the viability index values <1, especially, at a high concentration (200 μg/mL AM) (Figure 2C). Thus, engineering alums into NPs, especially, in combination with phospholipid coating, may provide an effective way to enhance biological function and adjuvanticity of the Al-based VADS.

### 3.3. The Al-Based Carriers Promote APC Activation and Engender Lysosome Escape

**Figure 3:**
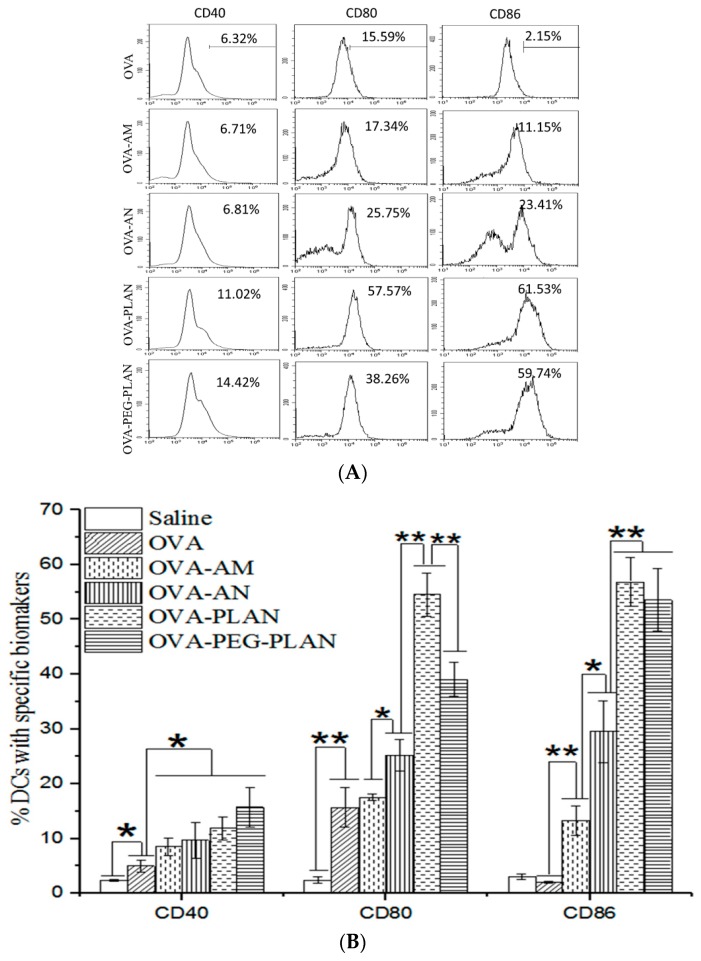

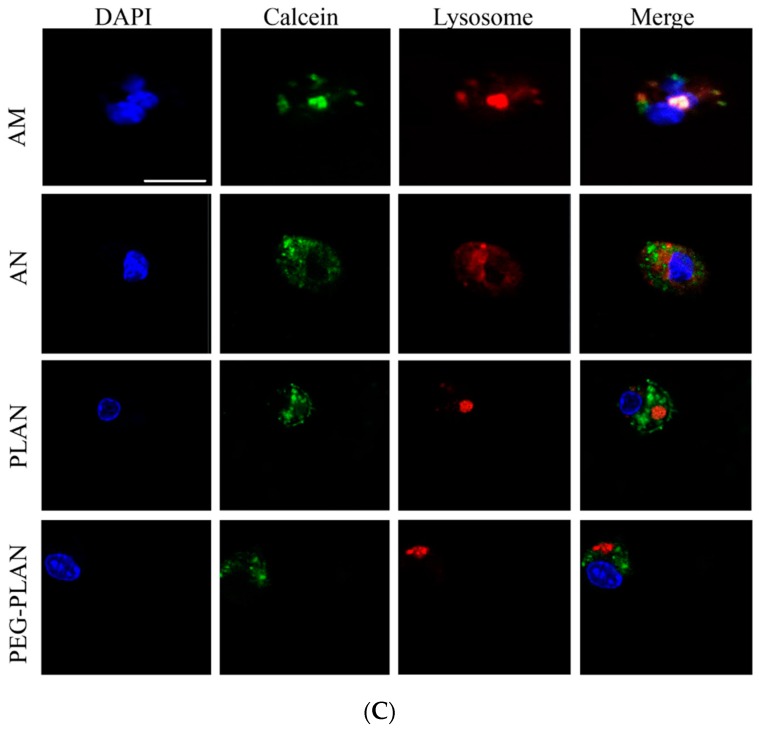
Activation of APCs stimulated by the Al-based VADSs and lysosome escape of the Al-based VADSs. (**A**,**B**) The expression levels of activation biomarkers (CD40, CD80, and CD86) on BMDCs after stimulation with the Al-based VADSs were obtained by flow cytometry assay of the cells stained with fluorescent-labeled Abs. (* *p*-value < 0.05, ** *p*-value < 0.01). (**C**) Intracellular locations of calcein-loaded Al-VADSs in APCs with 4′,6-diamidino-2-phenylindole (DAPI)-labeled nucleus (blue) and LysoTrack-red labeled lysosomes (red) were mapped with laser scanning confocal microscopy (LSCM) (bar = 10 μm, applicable to all panels).

After uptake of Ags or stimulation by a VADS, APCs may be activated to express enhanced surface markers, such as CD40, CD80, and CD86, which are necessary for further self-maturation and Ag presentation to prime T cells for sponsoring full anti-Ag immunoresponses. Usually, CD80 (also called B7.1) and CD86 (also called B7.2) are highly expressed on activated APCs and work in tandem to constitute one of the dominant co-stimulatory pathways. They prime T cell responses when binding to their receptors CD28 on T cells but downregulate immunoresponses when binding to CTLA4 (cytotoxic T-lymphocyte-associated protein 4, an immune check point) [30]. CD40 is also an enhanced biomarker on activated APCs and has the capacity to induce high levels of the cytokine IL-12. IL-12 proves able to fulfill multiple immune functions, such as polarizing CD4+ T cells toward a T helper 1 (Th1) type, enhancing proliferation of CD8+ T cells, activating NK cells, as well as upregulation of MHC-II and its co-stimulatory molecules CD80/CD86 [31]. Flow cytometry assay shows that the expressions of CD40, CD80, and CD86 on BMDCs were much more remarkably up-regulated by the stimulation with the Ag-loaded ANs, PLANs, or PEG-PLANs (Figure 3A,B) than with the AM-loaded or free Ags. Moreover, the levels of APC maturation markers highlighted PLANs as the most effective VADS in activating APC maturation to initiate the immunoresponses.

To track the intracellular location, the fluorescent agent calcein-loaded carriers were co-incubated with BMDCs. After that, cell lysosomes were labeled with Lyso-Tracker Red and nuclei with DAPI and then inspected by a LSCM. LSCM images reveal that ANs, PLANs, and PEG-PLANs taken up by the BMDCs existed mostly in the cytoplasm other than lysosomes (Figure 3C). Interestingly, LSCM inspection also disclosed that, in contrast to previous observations on alums [8,32,33], the AMs used in this study seemed to have also been internalized by APCs after co-incubation. The reason may be that the AMs are aggregates consisting of Al salt nano-crystals, which may be liberated from AMs and are thus picked up by the APCs. Nevertheless, the outcomes of the intracellular inspections suggest that the Al nanocarriers may possess the ability to engender lysosome escape, thus allowing the loaded Ags to avoid lysosomal degradation and maintain the immunostimulatory activity.

### 3.4. Vaccination of Mice with Al-Based Carriers Efficiently Delivers Agents to Lymph Node-Resident Immunocytes

**Figure 4:**
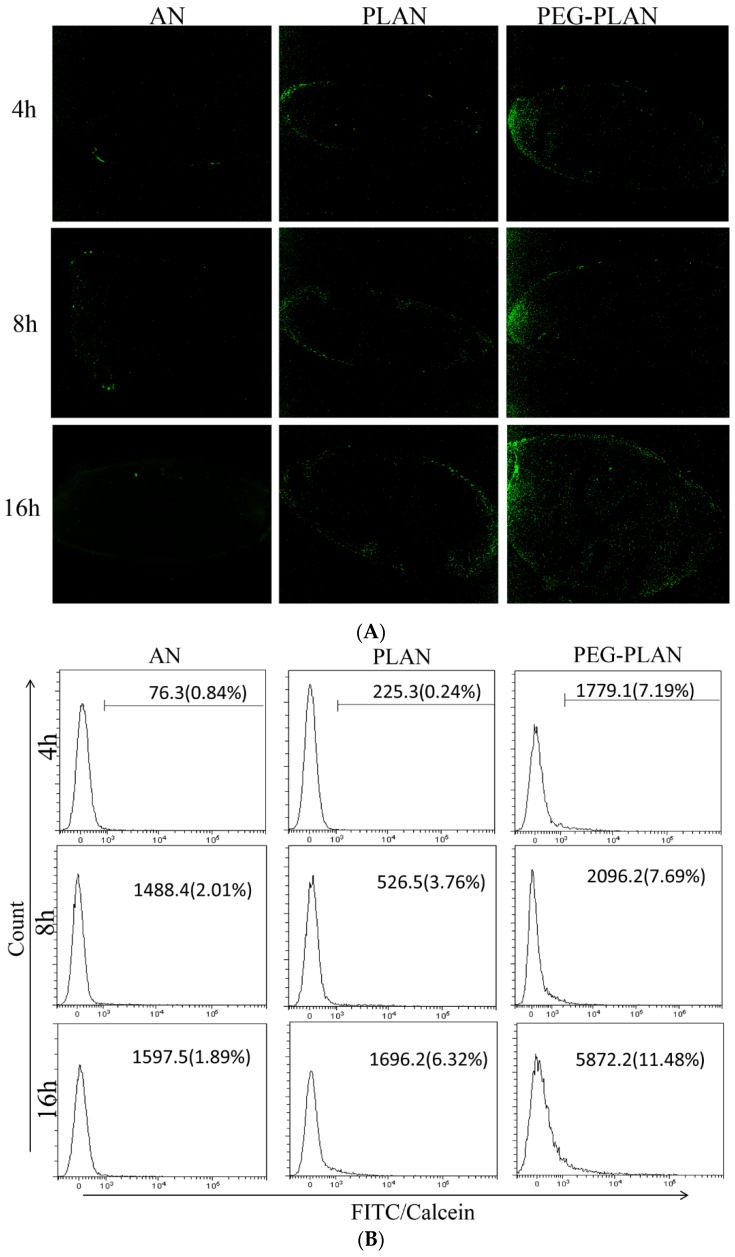

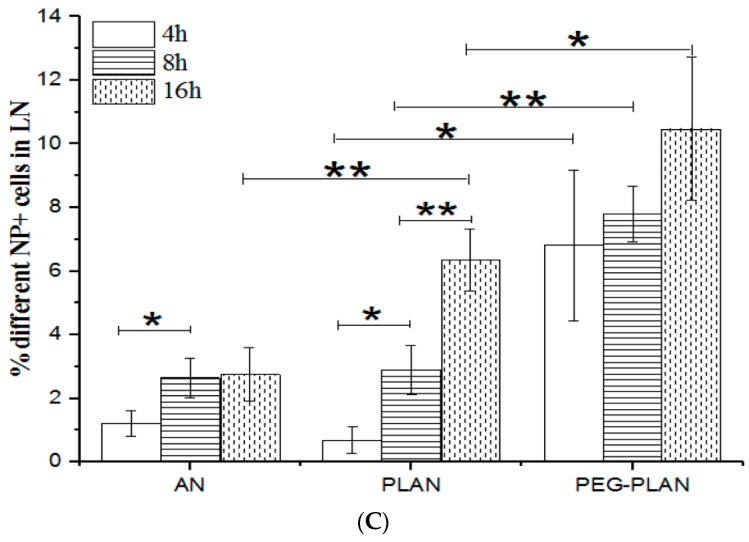
In vivo tracking of the calcein-labeled aluminum oxide nanoparticles (ANs), PLANs, and PEG-PLANs administered subcutaneously to mice at the lower extremity of lateral thigh (*n* = 3). (**A**) Fluorescent images of the cryo-sections of inguinal LNs separated from the mice that had been burdened with the Al-based carriers for different times. (**B**) The uptake frequencies of different calcein-labeled Al-VADSs by draining LN immunocytes at different times were estimated by flow cytometry. (**C**) The significant differences of cellular uptake of different Al-VADSs by draining LN immunocytes at different times were analyzed by *t*-test. (* *p*-value < 0.05, ** *p*-value < 0.01).

To track the Al-based nanocarriers in vivo, mice were subcutaneously vaccinated with the calcein-labeled nanocarriers (*n* = 3 for each formulation) at the lower extremity of lateral thigh. Then at different times after vaccination, mouse inguinal LNs responsible for draining the vaccination site were harvested either to make the OCT cryosections for LSCM imaging or to isolate immune cells for flow cytometry assay. The cryosection micrographs revealed that, 4 h after vaccination, a fraction of PLANs and a higher fraction of PEG-PLANs arrived in the inguinal LNs, as indicated by fluorescence intensity, which also showed that only a very small fraction of ANs approached the dLNs (Figure 4A). In line with these results, the flow cytometry assay demonstrated that, at different times after subcutaneous administration, PEG-PLANs were most efficiently picked up by dLN immunocytes (Figure 4B,C). However, by comparative dissection of LSCM images and flow cytometry results, it could be noticed that although 4 h after vaccination PLANs approached dLNs (Figure 4A), they were not taken up by the immunocytes within dLNs immediately (Figure 4B,C) but sometime later (before another 4 h). Given a reliable reproducibility of these results, a possible explanation is argued to be that the phospholipid cover of PLANs deterred their immediate recognition by APCs. In the case of ANs, the administered carriers were rarely detected of arrival at dLNs and internalization by LN immune cells. The reason for this may be that the strong affinity between cell membrane phospholipid and carrier Al engenders the administered ANs to be mostly taken up by the sentinel APCs that are patrolling around the vaccination site. These APCs after phagocytosis of ANs will be activated and then take about 16 h to travel from the vaccination site to the dLNs. During this relatively long-time journey, APCs may process the internalized fluorescent agents into small pieces, which do not emit fluorescence causing the activated APCs not to be detected in the dLNs.

Taken together, these outcomes suggest that PLANs after administration are prone to traffic to dLNs to facilitate immunocyte phagocytosis, while PEGylation greatly accelerates this process. This seems contradictory to the conventional cognition on PEGylation which is known to shelter NPs from recognition and clearance by immune cells.

### 3.5. Vaccination of Mice with Al-Based Carriers Elicits Robust Humoral Immunity with Th1-Biased Responses

**Figure 5:**
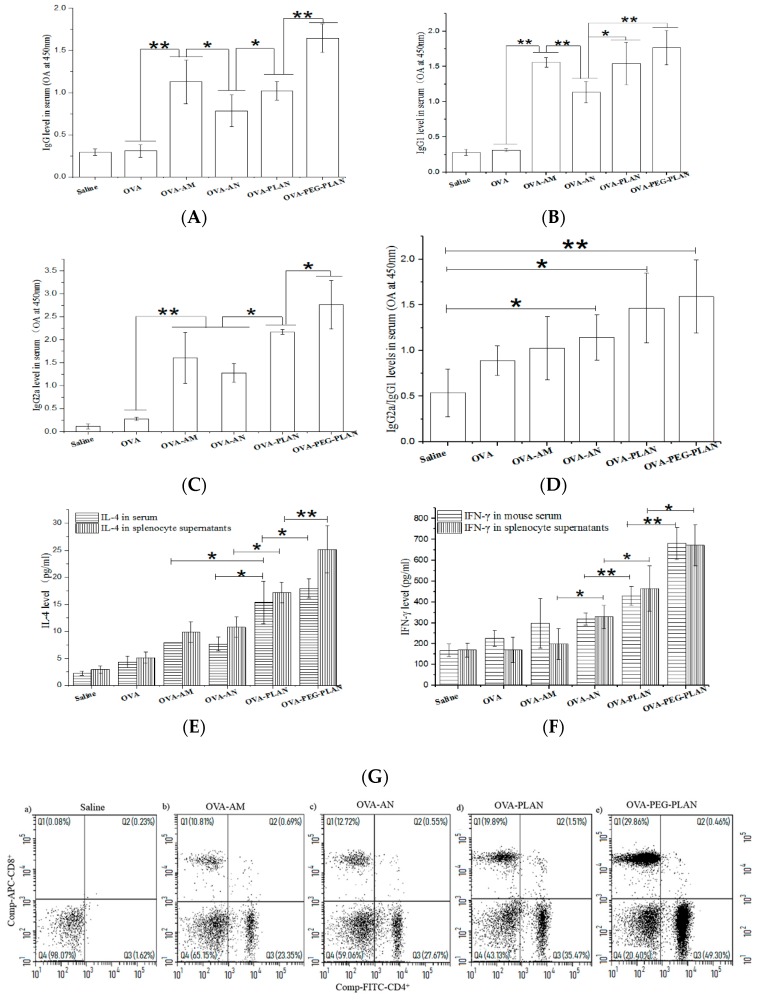
The Ag-specific immunity established in mice elicited by subcutaneous vaccination with Al-based carriers (*n* = 6). (**A**–**C**) The titer levels of IgG, IgG1, and IgG2a in mouse sera detected three weeks after immunization with different formulations. (**D**) The ratio of IgG2a/IgG1 in mice immunization with different formulations. (**E**,**F**) The levels of IL-4 and IFN-γ in mouse sera and blood of mice immunization with different formulations. (**G**) The frequency of CD4+ T and CD8+ T lymphocytes in spleens of mice immunization with different formulations. (* *p*-value < 0.05, ** *p*-value < 0.01).

The humoral and cellular immunity induced by Al-based carriers was evaluated by the determination of the levels of the Ag-specific Igs and CTLs in treated mice. As shown in Figure 5, compared to free Ags, the Al-based VADSs significantly enhanced the levels of mouse serum IgG, IgG1, and IgG2a (Figure 5A–C). Notably, whilst PLANs and PEG-PLANs elicited stronger humoral responses than AMs (alum), ANs with unknown reasons did not. However, by dissection of Ig data, it can be seen that the ratio of IgG2a/IgG1 in mice that received any type of the Al-based nanocarriers was all higher than that in mice that received the AMs (alum adjuvant) (Figure 5D). The results reflect that the Al-based nanocarriers, especially PLANs and PEG-PLANs, are in favor of triggering Th1-biased response, to sharply contrast alum which prefers Th2 response. In consistence with these results, the cytokine assays show that the Al-based nanocarriers triggered mice and their splenocytes to secret high levels of IL-4 as well as IFN-γ, whereas AMs (alum) only induced high levels of IL-4 (Figure 5E,F).

Also, flow cytometry assay of the splenocytes of vaccinated mice indicated that the Al-based carriers induced production of much higher frequency of CD8+ lymphocytes than did the micro-sized alum (Figure 5G), suggesting the Al-based nanocarriers as a VADS having the potential to elicit cellular immunity.

Summarily, the engineered aluminum NPs and, especially, the ones coated with phospholipid bilayers and PEG polymers are an effective VADS, able to induce robust immunoresponses toward the carried Ags and possessing the potential of eliciting strong humoral as well as cellular immunity.

## 4. Discussion

Previously, we made PLANs using Al(OH)_3_ NPs, which, however, were unstable polymorphic nanoaggregates and difficult to control in the size and shape, both of which are the important factor to influence the immunostimulatory functions [16]. Therefore, in this investigation, the very stable Al_2_O_3_ NPs with a size of 40 nm were used instead of Al(OH)_3_ NPs to prepare a novel version of PLANs and the PEGylated PLANs (PEG-PLANs) as a VADS. PEGylation of nanoparticles proves beneficial for the delivery of vaccines in several aspects, such as targeting dLNs, avoiding phagocytosis by non-professional APCs, and especially, enhancing product stability [26]. Enhancement of the stability of nanoparticles by PEGylation is argued to be caused by the steric stabilization effects of a highly hydrophilic polymer and has already been successfully employed for making the stealth liposome products [34], such as the marketed Doxil^®^, Onivyde^®^, and Onpattro^®^ [35]. Thus in this investigation, the PEG-PLANs were also engineered as a VADS for evaluation of their vaccine delivery effects. These Al-based nanocarriers, as well as the micro-sized alum (used as a control), were comparatively studied of the immunostimulatory activities through in vitro and in vivo experiments.

In comparison to alum, whilst ANs (Al_2_O_3_ NPs) showed only a few benefits in triggering immune system such as high safety, enhancing cellular uptake, and engendering lysosome escape, both PLANs and PEG-PLANs manifested many additional superiorities, including efficiently targeting dLNs, inducing Th1-biased immunoresponses, eliciting stronger humoral as well as cellular immunity. Notably, unlike alum with little ability to activate APC phagocytic response, perhaps due to its firm binding and disturbance of cell membranes [32], ANs can remarkably facilitate cellular uptake of their delivered cargos. A possible explanation for this is that the small size of ANs may engender the binding of ANs to APCs to exert only moderate membrane disturbance, which is insufficient to cause abortive phagocytic response but able to induce cellular internalization (Figure 2). In the case of PLANs, while the phospholipid coating has a high affinity to cell membranes, it also conceals the phosphophilicity of Al to nullify its membrane-binding/-sorting functions, thus facilitating cell internalization [16]. For PEG-PLANs, the enhanced cellular phagocytosis is argued to be attributed to the selective adsorption by PEG of interstitial proteins, rather than clusterin. The reason is that clusterin has recently proven able to form the specific protein corona surrounding NPs that accounts for the generation of stealth effects to abolish monocyte phagocytosis [36].

The intracellular investigations showed that the Al-nanocarriers engineered in this study after cellular internalization may manage lysosome escape, allowing Ags to avoid the destructive degradation by lysosomal enzymes. Lysosome escape may also render the delivered Ags the opportunity for cross-presentation and the downstream display of MHC-1-Ag-epitopes, favoring the Th1-biased responses and cellular immunity [37]. By comparison, micro-sized alum can hardly be internalized by APCs and thus has no chance to affect organelles and intracellular pathways, e.g., to engender lysosome escape and cross-presentation for Ags. As a result, a majority of Ags delivered by alum is subjected to lysosomal degradation and even extracellular destruction, thus compromising immunostimulatory effects while missing Th1 responses. This is in consistence with the well-known fact that the alum-adjuvanted vaccines generally induce recipients to produce predominantly the anti-Ag Abs over CTLs [9]. Although recently it was reported that injection of alum alone with a specific protocol stimulated non-specific anti-tumor CTL responses [13], the results needs further verification in both wide utility and practical feasibility.

This study also confirmed that small size and coating allow both PLANs and PEG-PLANs after topical administration to smoothly traffic to the dLNs, wherein the Al-based nanocarriers efficiently target and activate different populations of the immunocytes that are densely compartmentalized within dLNs. These immunocytes include DCs, murine macrophages (MPs), as well as follicular B cells and, upon activation, will immediately trigger T lymphocytes to orchestrate together for sponsoring the anti-Ag responses [38]. By comparison, the micron-sized alum after topical administration are trapped in the vaccination site, due to binding to cells as well as due to their large size, and therefore, will mainly be picked up by sentinel APCs, including skin-resident MPs, DCs, and Langerhans cells (LCs). However, only DCs and LCs can manage to migrate via the afferent lymphatics to the dLNs [39], wherein they mature and trigger T lymphocytes, thus lowering vaccination efficiency as a result of MP consumption of Ags without participation in the following reactions. Moreover, owing to being stranded in local site, the alum-carried Ags were mostly denied of the opportunity to access follicular B cells, whose activation, notably, is necessary for production of anti-Ag Abs, thus leading to inefficient vaccination [40]. By contrast, the Al-based nanocarriers, especially, PLANs and PEG-PLANs, as a VADS are capable of targeting and activating multiple populations of the dLN immune cells, which orchestrate together to efficiently initiate the Ag-specific immunoresponses for establishing immunity against pathogens bearing identical Ags. PEG-liposomes have also been employed by many researchers for delivery of vaccines for targeting dLNs [26,35]. For example, Perrie’s group prepared the PEGylated cationic nanoliposomes as a VADS and observed that the PEGylation engendered the cationic nanocarriers to target mouse dLNs as a result of the passive drainage. However, compared to the un-PEGylated counterparts, the PEGylated liposomes produced deceased depot effects while inducing lowered production of IgG2b and IFN-γ but elevated IL-5, suggesting the PEGylated cationic liposomes may favor triggering the Th2-biased immunoresponses [41]. By contrast, PEG-PLANs showed a great promise in stimulating the Th1/Th2 balanced reactions, as indicated by the high levels of IFN-γ and IgG2a and the high fraction of CD8+ T lymphocytes produced in mice immunized with PEG-PLANs versus PLANs.

## 5. Conclusions

In this investigation, the aluminum oxide NPs were coated with phospholipid bilayers to form stable PLANs, which were also further PEGylated to form PEG-PLANs. Both PLANs and PEG-PLANs are a stable VADS that can facilitate APC phagocytosis, engender lysosome escape for cross-presentation for Ags. After subcutaneous administration, PLANs and PEG-PLANs also can effectively target immune cells within draining lymph nodes, possessing potential to elicit robust humoral and cellular immunity. Thus, these aluminum oxide-based nanocarriers possess big advantages over conventional adjuvant alum and may provide an alternative VADS for making effective vaccines against pathogens.

## Figures and Tables

**Table 1:** The size (mean diameter ± SD) and distribution (PDI), zeta potential (mean ζ ± SD)) and association efficiency (AE) for OVA of the ANs, PLANs, and PEG-PLANs (*n* = 3).

Sample	Size ± SD (nm)	PDI	Zeta ± SD (mV)	AE ± SD (%) ^1^
AN	28 ± 0.4	0.28	−35.3 ± 1.36	77.9 ± 15.7
PLAN	33 ± 4.9	0.29	−25.7 ± 1.48	-
PEG-PLAN	31 ± 4.0	0.24	−28.4 ± 2.16	-

^1^ AEs of PLANs or PEG-PLANs made with the OVA-loaded ANs did not show any change after surface coating.

## References

[1] Di Pasquale A., Preiss S., Tavares Da Silva F., Garcon N. (2015). Vaccine Adjuvants: from 1920 to 2015 and Beyond. Vaccines.

[2] Shah R.R., Hassett K.J., Brito L.A. (2017). Overview of Vaccine Adjuvants: Introduction, History, and Current Status. Methods Mol. Biol..

[3] Levine M.M. (2011). “IDEAL” vaccines for resource poor settings. Vaccine.

[4] Barberis I., Myles P., Ault S.K., Bragazzi N.L., Martini M. (2016). History and evolution of influenza control through vaccination: From the first monovalent vaccine to universal vaccines. J. Prev. Med. Hyg..

[5] Moyle P.M., Toth I. (2013). Modern subunit vaccines: Development, components, and research opportunities. ChemMedChem.

[6] Wang T., Wang N. (2016). Preparation of the Multifunctional Liposome-Containing Microneedle Arrays as an Oral Cavity Mucosal Vaccine Adjuvant-Delivery System. Methods Mol. Biol..

[7] Wang N., Wang T. (2016). Preparation of Multifunctional Liposomes as a Stable Vaccine Delivery-Adjuvant System by Procedure of Emulsification-Lyophilization. Methods Mol. Biol..

[8] Wen Y., Shi Y. (2016). Alum: An old dog with new tricks. Emerg. Microbes Infect..

[9] Hem S.L., HogenEsch H. (2007). Relationship between physical and chemical properties of aluminum-containing adjuvants and immunopotentiation. Expert Rev. Vaccines.

[10] Li H., Li Y., Jiao J., Hu H.M. (2011). Alpha-alumina nanoparticles induce efficient autophagy-dependent cross-presentation and potent antitumour response. Nat. Nanotechnol..

[11] Li X., Hufnagel S., Xu H.Y., Valdes S.A., Thakkar S.G., Cui Z.R., Celio H. (2017). Aluminum (Oxy)Hydroxide Nanosticks Synthesized in Bicontinuous Reverse Microemulsion Have Potent Vaccine Adjuvant Activity. Acs Appl. Mater. Interfaces.

[12] Didierlaurent A.M., Morel S., Lockman L., Giannini S.L., Bisteau M., Carlsen H., Kielland A., Vosters O., Vanderheyde N., Schiavetti F. (2009). AS04, an aluminum salt- and TLR4 agonist-based adjuvant system, induces a transient localized innate immune response leading to enhanced adaptive immunity. J. Immunol..

[13] Wang B., Wang X., Wen Y., Fu J., Wang H., Ma Z., Shi Y., Wang B. (2015). Suppression of established hepatocarcinoma in adjuvant only immunotherapy: Alum triggers anti-tumor CD8+ T cell response. Sci. Rep..

[14] Orr M.T., Khandhar A.P., Seydoux E., Liang H., Gage E., Mikasa T., Beebe E.L., Rintala N.D., Persson K.H., Ahniyaz A. (2019). Reprogramming the adjuvant properties of aluminum oxyhydroxide with nanoparticle technology. NPJ Vaccines.

[15] Zhao Q., Sitrin R. (2001). Surface phosphophilicity of aluminum-containing adjuvants probed by their efficiency for catalyzing the P–O bond cleavage with chromogenic and fluorogenic substrates. Anal. Biochem..

[16] Wang T., Zhen Y.Y., Ma X.Y., Wei B., Wang N. (2015). Phospholipid Bilayer-Coated Aluminum Nanoparticles as an Effective Vaccine Adjuvant-Delivery System. Acs Appl. Mater. Interfaces.

[17] Ishida T., Iden D.L., Allen T.M. (1999). A combinatorial approach to producing sterically stabilized (Stealth) immunoliposomal drugs. FEBS Lett..

[18] Hamann S., Kiilgaard J.F., Litman T., Alvarez-Leefmans F.J., Winther B.R., Zeuthen T. (2002). Measurement of Cell Volume Changes by Fluorescence Self-Quenching. J. Fluoresc..

[19] Wang N., Wang T., Zhang M., Chen R., Niu R., Deng Y. (2014). Mannose derivative and lipid A dually decorated cationic liposomes as an effective cold chain free oral mucosal vaccine adjuvant-delivery system. Eur. J. Pharm. Biopharm..

[20] Jangle R.D., Galge R.V., Patil V.V., Thorat B.N. (2013). Selective HPLC method development for soy phosphatidylcholine Fatty acids and its mass spectrometry. Indian J. Pharm. Sci..

[21] Bradford M.M. (1976). A rapid and sensitive method for the quantitation of microgram quantities of protein utilizing the principle of protein-dye binding. Anal. Biochem..

[22] Zuo S.S., Lundahl P. (2000). A micro-Bradford membrane protein assay. Anal. Biochem..

[23] Lutz M.B., Kukutsch N., Ogilvie A.L., Rossner S., Koch F., Romani N., Schuler G. (1999). An advanced culture method for generating large quantities of highly pure dendritic cells from mouse bone marrow. J. Immunol. Methods.

[24] Zhang X., Goncalves R., Mosser D.M. (2008). The isolation and characterization of murine macrophages. Curr. Protoc. Immunol..

[25] Paillard A., Hindre F., Vignes-Colombeix C., Benoit J.P., Garcion E. (2010). The importance of endo-lysosomal escape with lipid nanocapsules for drug subcellular bioavailability. Biomaterials.

[26] Wang N., Zhen Y., Jin Y., Wang X., Li N., Jiang S., Wang T. (2017). Combining different types of multifunctional liposomes loaded with ammonium bicarbonate to fabricate microneedle arrays as a vaginal mucosal vaccine adjuvant-dual delivery system (VADDS). J. Control. Release.

[27] Zhen Y.Y., Wang N., Gao Z.B., Ma X.Y., Wei B.A., Deng Y.H., Wang T. (2015). Multifunctional liposomes constituting microneedles induced robust systemic and mucosal immunoresponses against the loaded antigens via oral mucosal vaccination. Vaccine.

[28] Wang N., Wang T., Zhang M., Chen R., Deng Y. (2014). Using procedure of emulsification-lyophilization to form lipid A-incorporating cochleates as an effective oral mucosal vaccine adjuvant-delivery system (VADS). Int. J. Pharm..

[29] Amrhein V., Greenland S., McShane B. (2019). Scientists rise up against statistical significance. Nature.

[30] Dilioglou S., Cruse J.M., Lewis R.E. (2003). Function of CD80 and CD86 on monocyte- and stem cell-derived dendritic cells. Exp. Mol. Pathol..

[31] Ma D.Y., Clark E.A. (2009). The role of CD40 and CD154/CD40L in dendritic cells. Semin. Immunol..

[32] Flach T.L., Ng G., Hari A., Desrosiers M.D., Zhang P., Ward S.M., Seamone M.E., Vilaysane A., Mucsi A.D., Fong Y. (2011). Alum interaction with dendritic cell membrane lipids is essential for its adjuvanticity. Nat. Med..

[33] Marichal T., Ohata K., Bedoret D., Mesnil C., Sabatel C., Kobiyama K., Lekeux P., Coban C., Akira S., Ishii K.J. (2011). DNA released from dying host cells mediates aluminum adjuvant activity. Nat. Med..

[34] Allen T.M., Cullis P.R. (2013). Liposomal drug delivery systems: From concept to clinical applications. Adv. Drug Deliv. Rev..

[35] Wang N., Chen M., Wang T. (2019). Liposomes used as a vaccine adjuvant-delivery system: From basics to clinical immunization. J. Control. Release.

[36] Schottler S., Becker G., Winzen S., Steinbach T., Mohr K., Landfester K., Mailander V., Wurm F.R. (2016). Protein adsorption is required for stealth effect of poly(ethylene glycol)- and poly(phosphoester)-coated nanocarriers. Nat. Nanotechnol..

[37] Van Endert P. (2016). Intracellular recycling and cross-presentation by MHC class I molecules. Immunol. Rev..

[38] Gutjahr A., Phelip C., Coolen A.L., Monge C., Boisgard A.S., Paul S., Verrier B. (2016). Biodegradable Polymeric Nanoparticles-Based Vaccine Adjuvants for Lymph Nodes Targeting. Vaccines.

[39] Tamoutounour S., Guilliams M., Sanchis F.M., Liu H., Terhorst D., Malosse C., Pollet E., Ardouin L., Luche H., Sanchez C. (2013). Origins and functional specialization of macrophages and of conventional and monocyte-derived dendritic cells in mouse skin. Immunity.

[40] Zabel F., Kundig T.M., Bachmann M.F. (2013). Virus-induced humoral immunity: On how B cell responses are initiated. Curr. Opin. Virol..

[41] Kaur R., Bramwell V.W., Kirby D.J., Perrie Y. (2012). Pegylation of DDA:TDB liposomal adjuvants reduces the vaccine depot effect and alters the Th1/Th2 immune responses. J. Control. Release.

